# Substitution of raw lucerne with raw citrus lemon by-product in silage: *In vitro* apparent digestibility and gas production

**DOI:** 10.3389/fvets.2022.1006581

**Published:** 2022-11-03

**Authors:** Maghsoud Besharati, Valiollah Palangi, Abdelfattah Zeidan Mohamed Salem, Pasquale De Palo, Jose Manuel Lorenzo, Aristide Maggiolino

**Affiliations:** ^1^Department of Animal Science, Ahar Faculty of Agriculture and Natural Resources, University of Tabriz, Ahar, Iran; ^2^Department of Animal Science, Agricultural Faculty, Ataturk University, Erzurum, Turkey; ^3^Department of Pharmacology, Anesthesia and Analgesia, Faculty of Veterinary Medicine and Animal Science, Autonomous University of the State of Mexico, Toluca, Mexico; ^4^Department of Veterinary Medicine, University of Bari A. Moro, Valenzano, Italy; ^5^Centro Tecnológico de la Carne de Galicia, Parque Tecnológico de Galicia, Ourense, Spain; ^6^Universidade de Vigo, Área de Tecnoloxía dos Alimentos, Facultade de Ciencias de Ourense, Ourense, Spain

**Keywords:** lucerne, lemon pomace, silage, *in vitro* apparent digestibility, gas production

## Abstract

Fruit pomace addition to lucerne silage could rapidly reduce silage pH creating an acidic environment and thus maybe preventing spoilage. However, the purpose of this study was to investigate the effect of different rates of inclusion of citrus lemon by-products on lucerne. In this study, the following five different treatments were prepared: L0 (control) with 100% lucerne; L25 (75% lucerne with 25% lemon pomace); L50 (50% lucerne with 50% lemon pomace); L75 (25% lucerne with 75% lemon pomace); and L100 (100% lemon pomace). After ensiling, the chemical composition, nutritive value, stability, *in vitro* apparent digestibility, and gas production of silage were determined. The dry matter (DM) content was higher for lemon pomace substitution equal to or exceeded 50% (*P* < 0.01). Crude protein, on the contrary, decreased (*P* < 0.01) over the same percentage of substitution. The L100 and L75 treatments showed higher DM apparent disappearance rate and lower (*P* < 0.05) crude protein and neutral detergent fiber apparent degradation rate *vs*. L0. Lemon pomace could be used at high inclusion level in lucerne silage, allowing the preservation of this by-product all the year, improving some chemical silage characteristics, and reducing proteolytic processes that usually happen on lucerne silage. Moreover, the *in vitro* apparent digestibility and gas production results showed that a partial substitution of lucerne with lemon pomace is able to improve silage digestibility.

## Introduction

Considering the growth of the human population in the last decades and the consequent increase in food demand, there is also a proportional growth in waste production and environmental impact (1). A vegetal-based food production chain has a central role in this context due to the generation of wastes during harvesting and/or processing (1). These by-products are useful resources and may play a pivotal role in different applications, in particular ruminant feeding (2). In fact, these waste products can be used also as ingredients in ruminant diets, aiming to reduce their environmental impact, reducing the food/feed competition, and land use (3, 4).

Citrus pomace (5, 6), tomato pomace (7, 8), apple pomace (9, 10), sugarcane pomace (11), grape pomace (12), and pistachio hulls (13) are identified as potential sources for livestock feed among the by-products of agricultural industries. Among these by-products, lemon pomace is an important ingredient for ruminant diets due to its low cost, which can reduce the cost of ruminant feeds (14). Moreover, the skin and seeds of lemons contain essential oils that can prevent the growth of some pathogenic microorganisms in the fermentation process of silage, also preventing energy loss in the form of methane and so improving fermentation efficiency. Although lemon pomace can be used fresh as a ration ingredient, the seasonality of its production, combined with inadequate storage techniques, can lead to nutrient losses and spoilage processes (15). However, silage treatment is an appropriate method to preserve vegetal by-products for posterior consumption, allowing farmers to use it throughout the year (16).

Silages are popular and useful components of ration in modern livestock operations (17). *Medicago sativa* L. (lucerne) is an important component of the ruminant ration. It plays an important role in ruminant feeding strategies due to its high palatability, as well as its high content of proteins, nitrogenous compounds, minerals, and vitamins (18, 19). Consequently, ensiling lucerne became quite common and widespread in humid regions (17). Nevertheless, it is challenging to improve lucerne silage quality because of the lack of an efficient fermentation process, as several issues have been reported: lack of water-soluble carbohydrates (WSC), high buffering capacity (20), vulnerability to Clostridia under adverse fermentation conditions (21), empty stem, and low dry matter (DM). Since about 30–40 years ago, carbohydrate and bacterial additive sources have been used to improve silage quality, and given that carbohydrate sources can stimulate fermentation but cannot prevent proteolysis, as a result, heterolactic fermentation takes place and leads to pH decrease (22). Extensive proteolysis of lucerne during silage fermentation is a prevalent problem (23). Several attempts have been done to reduce proteolysis in ensiled forage, mainly with application of chemical additives such as formic acid (24) and tannins (25, 26) or with biological additives such as bacteria strains and enzymes (27). Polyphenols have been shown to reduce both proteolysis and lipolysis in silage by deactivating specific enzymes or through the formation of protein–phenol–lipid complexes (28).

Therefore, the simultaneous use of microbial additives and a suitable source of carbohydrates in silage could produce better fermentation and higher nutritional value. Pre-drying and air-drying are other techniques applied to optimize the DM content of the forage before silage. However, very low DM silage is often associated with an increase in effluent production and Clostridium fermentation. Moreover, high DM silage is not well compressed and greatly reduces aerobic stability. The optimal DM for lucerne silage depends on the type of silage structure, environmental, and management conditions (29).

However, the objective of this study was to evaluate whether lemon pomace addition to lucerne silage gives a lower starting and ending silage pH, thus preventing the growth and proliferation of spoilage bacteria species. Starting from this hypothesis, the purpose of this study was to investigate the effect of mixing different levels of lemon pomace on lucerne silage characteristics (chemical composition, nutritive value, stability, *in vitro* digestibility, and gas production) intended for ruminant feed.

## Materials and methods

### Preparation of laboratory-scale silos

The whole second-cut lucerne (at the flowering stage) was harvested from a single farm (Ahar town) leaving a stubble of 5 cm and wilted to a DM of 250 g/kg of fresh weight (FW) (analysis reported below). Lemon pomace was obtained from a local juice processing industry (Marand town). Both wilted lucerne and lemon pomace were chopped manually by a paper cutter to nearly 2–3-cm in length. There were five different treatments in the ensiling trial: L0 (control) with 100% lucerne; L25 (75% lucerne with 25% lemon pomace); L50 (50% lucerne with 50% lemon pomace); L75 (25% lucerne with 75% lemon pomace); and L100 (100% lemon pomace), all ratios calculated on FW basis. The lucerne and lemon pomace were weighted according to the proportion described for each experimental trial and mixed by hand before packing into silos. Each mixture of each experimental treatment and for each silo was sampled (250 g) and immediately frozen (−20°C) in sealed vacuum plastic bags until further chemical analysis (chemical composition reported in Table 1).

**Table 1:** Chemical composition of raw materials before ensiling.

	**L0**	**L25**	**L50**	**L75**	**L100**
pH	6.64	6.06	5.45	4.75	4.12
DM^a^ (g/kg)	322.6	326.3	331.8	336.4	346.8
CP^b^ (g/kg DM)	175.2	152.5	127.5	102.3	76.8
Ash	104.2	103.5	100.5	94.5	92
EE^c^ (g/kg DM)	81.1	80.6	80.7	78.6	77.4
NDF^d^ (g/kg DM)	310.1	302.5	282.5	267.6	247.4
ADF^e^ (g/kg DM)	259.6	241.6	220.9	194.8	171
WSC^f^ (g/kg DM)	38.6	45.6	52.7	58.1	62
Lactic acid (g/kg DM)	5.77	5.55	5.30	5.06	4.81

^a^DM, dry matter; ^b^CP, crude protein, ^c^EE, ether extract; ^d^NDF, neutral detergent fiber; ^e^ADF, acid detergent fiber; ^f^WSC, water-soluble carbohydrates.

The silos were laboratory PVC (polyvinyl chloride) tubes with a height of 90 cm and a diameter of 10 cm with the volume of about 7 L. The silage density was calculated as the ratio between the ensiled material and silo volume (kg/m^3^), and it was on an average value of 650 g/L, and all silos were sealed with a screw cap and stored at room temperature (17–20° C) for 60 days. Each treatment had six different silos, and each silo was considered one experimental unit.

### Chemical analysis

Samples were analyzed before ensiling, after ensiling (excluding the first 15 and the last 15 cm of each silo), and after *in vitro* digestion in triplicate.

Dry matter was determined according to standard procedures [(30), method 930.15]. Ash was determined following standard procedures [(30), method 942.05] using a muffle furnace at 550°C for 16 h. Fat was determined using the Soxhlet extraction procedure (29, method 991.36), and crude protein (CP) was determined by Kjeldahl N × 6.25 procedures (24) [(30), method 968.06]. Neutral detergent fiber (NDF) and acid detergent fiber (ADF) were determined with the ANKOM fiber analyzer according to Van Soest et al. (31) considering the correction for residual acid-insoluble ash. Sodium sulfite was added to the solution for NDF determination. The WSC content was determined as described by McDonald et al. (32).

The volatile fatty acids (VFA) concentration was carried out using the method described by Ke et al. (23). At the opening, 20 g of FW from each silo was sampled and placed in a blender jar. Distilled water was added reaching a volume of 200 mL, and then, the samples were macerated for 30 s in a high-speed blender and filtered through four layers of medical gauze. The filtrate pH was measured immediately. After acidification with 7.14 M H_2_SO_4_, the filtrate was centrifuged for 15 min at 10,000 × g and filtered with a 0.45-μm dialyzer. Lactic acid, acetic acid, propionic acid, and butyric acid were analyzed by high-performance liquid chromatography (HPLC, KC-811 column, Shodex; Shimadzu; Japan; oven temperatures 25°C; flow rate /min; SPD 210 nm).

The ammonia nitrogen was then recorded as described by (33). One milliliter of 250 g/L (w/v) trichloroacetic acid (TCA) was added to 4 ml of the previously obtained filtrate. Then, the solution was left at room temperature for 1 h to precipitate the protein. After this, the solution was centrifuged at 4°C, 18,000 × g for 15 min, and the supernatant fluid was analyzed for ammonia nitrogen (NH_3_-N).

Fleigh points (FPs) for silage quality were calculated according to Lashkari et al. (34) with the following equation: FP = 220 + [(2 × % DM)−15] - [40 × pH]. Silage quality was assessed according to the following criteria: very good (85–100), good (60–85), moderate (55–60), satisfying (25–55), and bad quality/worthless (< 25).

### *In vitro* apparent degradability and gas production

Rumen fluid was obtained from a total of 48 animals (Limousine beef, 15 months old, reared in the same farm, fed with the same ration) immediately after slaughtering procedures. Rumen fluid sampling procedures were conducted, as described by Maggiolino et al. (35). Rumen fluid was collected in thermos flasks (previously filled with distilled water at 39°C to avoid thermal shock to the rumen fluid), insufflated with CO_2_ into the headspace to ensure an anaerobic microenvironment, and took to the laboratory within 1 h. After transport, the top layer of ruminal content was discarded, and the remaining portion was mixed and blended under a CO_2_ headspace for about 1 min to remove any additional particles and/or attached organisms. The combined fluid and contents were strained through six layers of cheesecloth to form the inoculum for the *in vitro* fermentation, which was conducted for 48 h using the Daisy II incubator system (ANKOM Tech., Fairport, NY, USA). The unit consisted of four incubation vessels with a capacity of 2 L each. Each vessel contained 1.6 L of buffer solution, 400 mL of rumen liquor, and 25 nylon filter bags (ANKOM F57, ANKOM Tech., Fairport, NY, USA). The buffer solution consisted of 1.33 L of buffer A (KH_2_PO_4_, 10.0 g/L; MgSO_4_ H_2_O, 0.5 g/L; NaCl, 0.5 g/L; CaCl_2_ H_2_O, 0.1 g/L; and urea, 0.5 g/L) and 266 mL of buffer B (15.0 g/L Na_2_CO_3_ and 1.0 g/L Na_2_S_7_H_2_O). This buffer was added to each digestion vessel, and the pH was adjusted to 6.8 (36). Each digestion for each sample was performed in duplicate (two vessels). Bags were rinsed with acetone and allowed to air-dry before drying at 100 °C for 24 h, after recording dry bag weight. They were used and filled with a total of 0.5 g of each sample of each silo (all samples were stored at −20 °C before analysis).

All samples were previously ground until the particle size reached a 4-mm screen using a hammer mill (Pulverisette 19, Fritsch GmbH, Laborgeratebau, Germany). After digestion, all bags were weighed again, and all analyses for nutrient parameters were determined after digestion. The percentage of weight difference for each parameter was calculated as reported by (12) as follows: (PB-PA)/PB, where PB is the quantity (g/kg) of the parameter in the samples before digestion and PA is the quantity (g/kg) of the parameter after digestion.

For gas and VFA analysis, an automated pressure transducer system (ANKOM Technology, Macedon, NY, USA) was used as described by Maggiolino et al. (35). The system was equipped with eight different 250-mL bottles, and the same buffer solutions for the *in vitro* digestion section were used. Each vessel received 133.3 mL of buffer A and 26.7 mL of buffer B. Then, 40 mL of rumen fluid was added. All samples (0.5 g) were pre-weighed into each vessel, and the headspace of each vessel was insufflated with CO_2_ for 2 min to ensure anaerobic conditions. The vessels were put in an oscillating water bath (39 °C with an oscillating frequency of 45/min), to reproduce movements like those found in the rumen. Digestion was simulated for a 48-h fermentation period. After this period, the vessels were removed from the water bath and placed into an ice bath, while gas samples were drawn into evacuated test tubes, as described by Trotta et al. (37). The gas samples were analyzed for methane production with gas chromatography (Agilent Technologies, Santa Clara, CA, USA) by using the total gas volume at standard temperature and pressure (38). Flasks were opened, and the pH was measured. Then, a 1-mL aliquot of the fermentation medium was combined in a 1.5-mL centrifuge tube with 0.1 mL of 500 g/L of metaphosphoric acid and 0.1 mL of 85 mM of 2-ethyl butyrate. The samples were centrifuged at 39,000 × g, at 23 °C for 15 min, and afterward, the tubes were processed for VFA concentrations (37) using a gas chromatograph equipped with a 2 m × 3 mm packed column (45.60 Carboxen 1000, Supelco, Inc., Bellefonte, PA, USA) and flame ionized detector (FID) (Agilent Technologies, Inc., Santa Clara, CA, USA). For determination of ammonia nitrogen, 2 Ml of fluid and 2 of mL trichloroacetic acid solution (10%, w/v) were mixed to deproteinize the samples and then centrifuged for 5 min at 1,500 × g. The supernatant (2 mL) was processed in order to measure the ammonia nitrogen concentration according to a spectrophotometric method (39).

### Statistical analysis

The obtained data were analyzed in a completely randomized statistical design according to the general linear model (GLM) procedure. There were six silos for each experimental trial, each one considered as one experimental unit. The dataset was tested for normal distribution and variance homogeneity (Shapiro–Wilk's test). Afterward, one-way ANOVA was performed to differentiate among the treatments the chemical composition, VFA content, *in vitro* digestion data, gas emission, and VFA emission results after digestion. The ANOVA was performed according to the following model:


$$\begin{array}{l} Y_{\text{ijk}}=\mu +\alpha_{\text{i}}+T_{\text{j}}+\;\varepsilon_{\text{ijk}} \end{array}$$

where Y_ijk_ represents all previously cited patterns as dependent variables, μ is the overall mean, α_i_ is the ith silo random effect (i = 1,…6), T_j_ is the effect of the jth mixing treatment (j = 1, …5), and ε_ijk_ is the error term. Significance was set at P < 0.05, and the results were expressed as means and mean standard error. All the analyses were performed using SAS software (40).

## Results

### Chemical composition

The silage pH decreased (*P* < 0.01) with the increasing rate of inclusion of lemon pomace. The DM content was higher (*P* < 0.01) for lemon pomace substitution equal to or superior to 50%. Crude protein, on the contrary, decreased (*P* < 0.01) over the same percentage of substitution, while ash and ether extract did not show any significant difference. The neutral detergent fiber concentration was higher in L100 compared with L0 (*P* < 0.05), while no differences were observed in neutral detergent fiber content. Lactic acid increased (*P* < 0.05) with the increasing proportion of lemon pomace, while the opposite trend was recorded for acetic acid (*P* < 0.05). The ammonia content decreased (*P* < 0.01) linearly with the increasing inclusion of lemon pomace, and the FP values increased (*P* < 0.05) as lemon pomace increased (Table 2).

**Table 2:** Chemical composition, volatile fatty acid production, and Fleigh point of different substitution treatments of lucerne with lemon pomace by-product after 60 days of ensiling.

	**L0**	**L25**	**L50**	**L75**	**L100**	**MSE**	***P*-value**
pH	5.85^A^	5.18^B^	4.30^C^	3.61^D^	3.39^D^	0.19	0.0068
DM (g/kg)	163.5^A^	178.0^A^	222.9^B^	239.6^B^	251.6^B^	10.8	0.0016
CP (g/kg of DM)	174.23^A^	144.58^A^	118.26^B^	89.62^Ca^	61.10^Cb^	9.66	0.0025
Ash	8.95	8.82	7.52	9.05	9.94	1.04	0.6291
EE (g/kg of DM)	72.35	71.25	70.65	71.65	68.35	6.58	0.4770
NDF (g/kg of DM)	302.1^a^	285.4	273.5	261.5	255.8^b^	12.8	0.0418
ADF (g/kg of DM)	251.25	260.58	272.85	281.54	274.65	42.54	0.4029
WSC (g/kg of DM)	4.56	3.59	3.95	5.32	4.68	0.12	0.5442
Lactic acid (g/kg of DM)	52.20^a^	53.21^a^	55.55	58.21^b^	61.84^b^	9.04	0.0229
Acetic acid (g/kg of DM)	32.32^a^	30.25	28.56	24.12^b^	23.58^b^	4.56	0.0391
NH_3_-N (g/kg of TN)	105.56^A^	82.24^B^	51.56^C^	30.54^D^	25.36^D^	11.21	0.0084
Fleigh point	37.7^a^	33.4^a^	77.58^b^	88.52^c^	99.72^d^	2.32	0.0107

DM, dry matter; CP, crude protein; EE, ether extract; NDF, neutral detergent fiber; ADF, acid detergent fiber; WSC, water-soluble carbohydrates; NH_3_-N, ammonia N; TN, total N; MSE, mean standard error.

L0, 0 lemon pomace; L25, 25% lemon pomace, L50, 50% lemon pomace; L75, 75% lemon pomace; L100, 100% lemon pomace.

Different letters in the same line correspond to statistical differences. A, B, C = P < 0.01; a, b, c = P < 0.05.

### *In vitro* apparent digestibility and gas production

The L100 and L75 showed higher DM apparent disappearance rate and lower (*P* < 0.05) crude protein and neutral detergent fiber apparent disappearance compared with L0 (Table 3). No differences in apparent digestibility were observed for ether extract and acid detergent fiber. The L75 and L100 treatments showed higher total VFA and acetic acid production compared with L0 (*P* < 0.05). Moreover, L100 showed lower (*P* < 0.05) nitrogen production compared with L0 (Table 4).

**Table 3:** Percentage of disappearance after 48-h *in vitro* digestion in different substitution treatments of lucerne with lemon pomace by-product after 60 days of ensiling.

	**L0**	**L25**	**L50**	**L75**	**L100**	**SEM**	***P*-value**
DM	45.40^a^	47.01	48.52	50.25^b^	51.81^b^	7.58	0.0412
CP	50.12^a^	48.45	47.65	46.52^b^	44.98^b^	6.26	0.0308
EE	34.54	35.25	34.27	36.14	32.58	5.87	0.7118
NDF	51.45^a^	48.54	49.25	46.05^b^	44.54^b^	5.28	0.0252
ADF	48.25	46.58	47.95	48.24	47.65	6.08	0.8118

DM, dry matter; CP, crude protein; EE, ether extract; NDF, neutral detergent fiber; ADF, acid detergent fiber, NFC, non-fiber carbohydrate.

L0, 0 lemon pomace; L25, 25% lemon pomace, L50, 50% lemon pomace; L75, 75% lemon pomace; L100, 100% lemon pomace.

Different letters correspond to statistical differences. a, b = P < 0.05.

**Table 4:** *In vitro* gas production after 48-h *in vitro* digestion in different substitution treatments of lucerne with lemon pomace by-product after 60 days of ensiling.

	**L0**	**L25**	**L50**	**L75**	**L100**	**MSE**	***P*-value**
Total VFA (mmol/L)	105.45^a^	108.78	112.45	120.84^b^	122.81^b^	6.91	0.0293
Acetic acid (mmol/L)	65.45^a^	66.87	70.54	72.45^b^	75.84^b^	4.09	0.0318
Propionic acid (mmol/L)	25.45	26.85	27.78	28.88	29.48	4.12	0.4749
Butyric acid (mmol/L)	15.44	16.41	15.65	18.15	19.14	3.85	0.0899
Methane (mmol/L)	16.45	16.85	15.18	17.22	18.37	0.85	0.4199
Nitrogen (NH3-N) (mmol/L)	25.25^a^	22.84	20.23	15.65^b^	15.87^b^	1.14	0.0158

VFA, volatile fatty acids; L0, 0% lemon pomace; L25, 25% lemon pomace, L50, 50% lemon pomace; L75, 75% lemon pomace; L100, 100% lemon pomace.

Different letters correspond to statistical differences. a, b, c = P < 0.05.

## Discussion

### Chemical composition

It is known that a successful silage fermentation process produces a rapid decline in pH values and a high concentration of lactic acid. In fact, successful ensiling depends on several factors such as the presence of suitable microflora, adequate substrate, as well as suitable physical environments able to develop desirable fermentation. Legume forages like lucerne usually do not generate good-quality silage due to their buffer capacity and their low WSC concentration (41). The substitution of lucerne with lemon pomace reduced the final pH due to the initial pH of the substrate and probably due to the modulated fermentation processes of the ensiled material. The use of carbohydrate sources in the lucerne silages decreased the pH and increased the lactic acid concentration. However, lucerne silage is one of the hard-to-store plants for storage (42), and effectively a detriment effect on silage quality can be observed in lucerne characterized by low WSC and DM content, as well as low population of lactic acid bacteria. In these cases, a non-lactate fermentation can occur, resulting in DM and nutrient losses (43).

One of the most important and common issues in ensiling lucerne is the extensive proteolysis during silage fermentation. In fact, a reduction in proteolysis during ensilage could improve the utilization of silage-N because the efficiency of rumen microbial-N synthesis may be improved by supplementing silage with protein-N rather than NPN (44). The metabolic processes during the forage's fermentation can alter the original nutritive value of the ensiled forage. True protein content declines, whereas soluble protein increases due to the proteolytic processes (45). However, these processes are usually extended in lucerne silage, with higher NH_3_-N production. Probably, as observed by Rodrigues et al. (46) the substitution of lucerne with lemon pomace can reduce the proteolysis processes, reducing the NH_3_-N production. It is not well known why adding fruit pomace, such as lemon pomace, could reduce proteolysis, but similar results were reported by Ke et al. (23) substituting lucerne with apple pomace. Many authors affirm that the polyphenols fraction of tannins could inhibit the proteolysis in ensiled forage (26, 47) through a different mechanism such as the deactivation of proteolytic enzymes and/or throughout the formation of protein–polyphenol complexes (28). Pomace in general, particularly lemon pomace, could be a rich source of antioxidant compounds such as phenolics (48), so the apparent proteolysis inhibition effect could be attributed to the high amount and variety in lemon pomace of polyphenols, and it is not currently well-known interaction with microbial communities and biochemical pathways.

### *In vitro* apparent digestibility and gas production

Feed digestibility represents one of the most important factors in the nutritional efficiency assessment of a ration. The DM apparent digestibility tended to increase with the increasing inclusion rate of lemon pomace, although the crude protein and neutral detergent fiber loss tended to decrease. It is interesting to highlight that the results show a greater microbial digestion efficiency on the DM, while it seems to lose efficiency on crude protein degradation. Lemon pomace, as previously reported, is richer in polyphenols compared with lucerne, and the final silage should be also characterized by higher polyphenols content. These substances are reported to have antimicrobial properties (49, 50) and had the ability to inhibit digestive processes by reducing rumen microbial fermentation throughout the alteration of some microorganisms' cytoplasmic membrane functions, bacterial cell wall, or nucleic acid synthesis (51). However, contrasting results are reported in the literature on this activity. In fact, some studies reported that the inclusion of some substrates rich in polyphenols can promote fiber and carbohydrate digestion efficiency and could increase the production of microbial mess (52), Moreover, higher digestibility could be attributed to the addition of highly digestible gluten by lemon pomace substitution (53). Some results are attributed to different diets test as well as to different microbial families, respectively, inhibited and/or stimulated (54, 55).

The total VFA production results were consistent with the *in vitro* apparent digestibility of DM (56), as both increased with the lemon pomace inclusion proportion. On the contrary, neutral detergent fiber and crude protein apparent digestibility decreased. It must be stated that a linear correlation between total VFA production and digestibility was not found. There is the possibility that microbial activity can be improved or limited by some active compounds (*i.e.*, with antioxidant activity as phenolic compounds), thus improving VFA production without directly increasing all feed nutrients digestibility. Probably, the greater carbohydrate availability from fruit pomace, such as lemon pomace, can result in higher digestibility of this fraction. The substrate able to improve butyric acid production is the dietary fiber, throughout different metabolic pathways as a result of bacterial fermentation, starting from glucose and, consequently, carbohydrate availability (57).

The NH_3_-N represents one of the major nutritive sources for microbial growth and microbial protein synthesis in the rumen (58). Its production rate is related to the dietary rumen protein degradation (59). Therefore, higher NH_3_-N values probably could be due to the increased protein digestibility. In our study, both apparent digestibility and protein content decreased with the substitution of lucerne with lemon pomace, justifying the tendency of NH_3_-N rate to decrease. The NH_3_-N is a key metabolite for nitrogen production, and its decrease is due to lower protein degradation in feed and consequently lower microbial protein synthesis (35), and this result shows that although lemon pomace can give more protein stability and lower proteolytic processes, there is a lower nutrient availability for microbial populations.

## Conclusion

Supplementation of lemon pomace could be used in high proportion in lucerne silage, giving the possibility to make it available all the year as feed and to improve some chemical silage patterns, reducing proteolytic processes that usually happen on lucerne silage. Partial substitution of lucerne with lemon pomace is able to improve silage digestibility, although more deep studies should be conducted to better understand the effects on rumen microbiota, considering that the use of a carbohydrate source is useful for bacterial population activity. However, the sustainability of a waste product as a feed, which is useful to reduce its environmental impact of the citrus industry and animal feeding, improves silage quality and promotes circular economy.

## Data availability statement

The raw data supporting the conclusions of this article will be made available by the authors, without undue reservation.

## Ethics statement

The animal study was conducted in accordance with the Declaration of Helsinki, and approved by the Institutional Review Board (or Ethics Committee) of University of Tabriz (protocol code Tabri-zU-300/10M-2021).

## Author contributions

MB and VP contributed to conceptualization, data curation, formal analysis, and writing—original draft. AS contributed to data curation and formal analysis. PD contributed to writing—review and editing. AM contributed to investigation and writing—original draft. JL contributed to funding acquisition, resources, project administration, and writing—review and editing. All authors contributed to the article and approved the submitted version.

## Funding

This work was supported by the University of Tabriz, International and Academic Cooperation Directorate, in the framework of the TabrizU-300 program.

## Conflict of interest

The authors declare that the research was conducted in the absence of any commercial or financial relationships that could be construed as a potential conflict of interest.

## Publisher's note

All claims expressed in this article are solely those of the authors and do not necessarily represent those of their affiliated organizations, or those of the publisher, the editors and the reviewers. Any product that may be evaluated in this article, or claim that may be made by its manufacturer, is not guaranteed or endorsed by the publisher.
